# Increasing leadership capacity for HIV/AIDS programmes by strengthening public health epidemiology and management training in Zimbabwe

**DOI:** 10.1186/1478-4491-7-69

**Published:** 2009-08-10

**Authors:** Donna S Jones, Mufuta Tshimanga, Godfrey Woelk, Peter Nsubuga, Nadine L Sunderland, Shannon L Hader, Michael E St Louis

**Affiliations:** 1 Division of Global Public Health Capacity Development (previously Division of International Health), Centers for Disease Control and Prevention, Atlanta, Georgia, USA; 2 MPH Programme, Department of Community Medicine, University of Zimbabwe Faculty of Medicine, Harare, Zimbabwe; 3 RTI International, Research Triangle Park, North Carolina, USA; 4 Global AIDS Program, Centers for Disease Control and Prevention, Atlanta, Georgia, USA; 5 HIV/AIDS Administration, DC Department of Health, Washington, DC, USA; 6 Coordinating Office for Global Health, Centers for Disease Control and Prevention, Atlanta, Georgia, USA

## Abstract

**Background:**

Increased funding for global human immunodeficiency virus prevention and control in developing countries has created both a challenge and an opportunity for achieving long-term global health goals. This paper describes a programme in Zimbabwe aimed at responding more effectively to the HIV/AIDS epidemic by reinforcing a critical competence-based training institution and producing public health leaders.

**Methods:**

The programme used new HIV/AIDS programme-specific funds to build on the assets of a local education institution to strengthen and expand the general public health leadership capacity in Zimbabwe, simultaneously ensuring that they were trained in HIV interventions.

**Results:**

The programme increased both numbers of graduates and retention of faculty. The expanded HIV/AIDS curriculum was associated with a substantial increase in trainee projects related to HIV. The increased number of public health professionals has led to a number of practically trained persons working in public health leadership positions in the ministry, including in HIV/AIDS programmes.

**Conclusion:**

Investment of a modest proportion of new HIV/AIDS resources in targeted public health leadership training programmes can assist in building capacity to lead and manage national HIV and other public health programmes.

## Background

The last several years have seen a remarkable increase in funding for global health [1-3]. Most of these new resources for global health come tightly linked to addressing specific disease problems, e.g. immunizable diseases or HIV/AIDS. Despite the important accomplishments of this approach, it has been increasingly recognized that this vertical funding and its accompanying structure does not automatically address and may worsen the underlying issues that severely reduce the capacity to respond to each disease [4-6]. The most critical constraint to effective response is weakened infrastructure and systems of public health. The rapid expansion of programmes in such contexts can easily lead to only short-term impacts and further weakening of public health infrastructure [6].

Many of these new resources may be wasted if human resource constraints are not addressed [7,8]. The clear challenges facing the public health workforce, particularly in developing countries, have been well documented [9-11]. This is a critical component of the global human resource crisis that is limiting the ability of the world to respond effectively to health crises [2,5]. The HIV/AIDS crisis in sub-Saharan Africa has exacerbated the problem, both by increasing the magnitude of the health crises and diminishing the number of available health workers [2,5,12]. Countries applying to the Global Fund to Fight AIDS, Tuberculosis and Malaria have consistently identified human resources as a top priority for health system strengthening [6].

In difficult environments, where few trained persons might be available, it has been very tempting for international organizations to hire away well-trained persons from national institutions for specific small projects, typically funded by nongovernmental organizations (NGOs) [7,13]. Because of the great resources for HIV/AIDS, AIDS programmes are perhaps at particular risk for this unintended consequence. This may lead to a successful small project but can inadvertently undermine the long-term goals of capacity strengthening and institution building. This has been especially true in the context of the HIV/AIDS epidemic in developing countries [2,11,13,14]. This problem has now been recognized and acknowledged by the donor community and the countries, which increasingly plan to better coordinate aid to support national health systems rather than focusing exclusively on disease-specific priorities[15,16].

Zimbabwe is one of the countries most severely affected by HIV/AIDS. The estimated HIV prevalence in 2003 was reported at 24.6% [17]. In 2000, increased funds for responding to the epidemic in Zimbabwe became available through the United States Centers for Disease Control and Prevention's (CDC) Global AIDS Program (GAP). Like many countries, Zimbabwe faced the problem of absorption capacity: limited capacity to translate new financial resources into effective programmes.

In particular, the number of persons trained for leadership and management of new HIV intervention programmes was insufficient. Inadequate remuneration for public health officials and faculty was leading to loss of staff, or to staff working extra jobs to compensate for poor public sector salaries, thus limiting time available both to perform public health tasks and train public health staff. A related problem, as alluded to above, was "internal brain drain" reflected by hiring of national public sector staff to work on internationally funded HIV projects, further draining the necessary coordinating capacity and infrastructure [7,13].

Zimbabwe had long recognized the need for locally trained public health professionals. A Masters in Public Health (MPH) programme using the applied epidemiology training programme model had been started in 1994 through support of the Public Health Schools without Walls (PHSWOW) Programme of the Rockefeller Foundation and has continued with support from CDC's Division of Global Public Health Capacity Development (DGPHCD) (formerly Division of International Health (DIH) [10,18-20]. PHSWOWs were developed as partnerships between ministries of health and universities. Applied epidemiology training programmes, also known as field epidemiology training programs (FETP) are designed to build human capacity in health service agencies by providing training in field epidemiology and other public health competences in the context of health service delivery systems [18,21,22].

The Zimbabwe MPH programme began in 1993 as a joint effort of the Ministry of Health and Child Welfare (MOH) and the Department of Community Medicine at the University of Zimbabwe (UZ). A CDC advisor was resident in Zimbabwe during 1994–1996. During 1994–2000, a total of 41 trainees graduated. Most trainees were physicians and nurses, but pharmacists, veterinarians, nutritionists, laboratorians and other health staff were trained.

The programme was well respected and well integrated into the public health system and the MOH, as indicated by the fact that the majority of graduates were employed within the national public health system at either national-level positions in MOH, as Provincial Medical Directors or as City Health Directors. However, with only four to eight graduates per year, the number of public health professionals still did not meet the country's needs. In addition, the MPH curriculum in Zimbabwe had not been updated in response to the emergence of the HIV/AIDS crisis. Despite the >50% national burden of disease attributable to HIV in Zimbabwe [23], the focus on HIV was limited, with only three of 41 MPH dissertation topics during 1994–2000 being HIV/AIDS-related.

When CDC GAP began to work in Zimbabwe in 2000, despite 10 years of Zimbabwean research and reports on HIV/AIDS, there was limited implementation of truly nationwide HIV prevention and treatment programmes to slow the epidemic and attenuate its impact. CDC GAP, together with the MOH, jointly developed specific goals related to HIV prevention and control for Zimbabwe. These included expansion of Prevention of Mother to Child Transmission of HIV (PMTCT), expansion of HIV testing capacity, improved understanding of the epidemic through better surveillance methods, behavioural interventions, improvement of care for opportunistic infections and introduction of antiretrovirals (ARV) throughout the country.

A key supporting strategy of CDC GAP was to build human capacity and strengthen the existing health institutions to provide the needed leadership in a sustainable way. This paper describes one specific collaborative effort, begun in 2001, that built on the strength and resources of many partners to respond more effectively to the HIV/AIDS epidemic by reinforcing a crucial training institution and thereby expanding production of epidemiologists and public health leaders.

## Methods

### Programme description

CDC GAP provided financial resources for a broad array of national HIV/AIDS programmes, including extensive in-country technical support. The University of Zimbabwe, Department of Community Medicine, already had faculty members and experience with running a successful field training-based MPH programme. The MOH had been providing the province-level posts and first-line mentorship during the critical field training period. In addition, and probably most important, the MOH was the major employer of MPH graduates, recruiting them into positions of responsibility and establishing this training programme as its main career-development conduit for senior public health leaders and managers. The Division of Global Public Health Capacity Development (DGPHCD) at CDC had long partnered with the UZ/MPH programme and had substantial experience in supporting applied epidemiology training in many other countries.

The partners shared a vision that programmatic HIV prevention and care goals could be met by strengthening public health systems and supporting specific areas of national human capacity development rather than by narrowly addressing only HIV programmatic needs.

This collaboration developed a number of goals for the programme:

• Strengthen the public health leadership training programme and increase its output.

• Increase the focus on training to produce public health leaders explicitly equipped to design and implement HIV intervention programmes.

• Increase the number of HIV and related positions in the MOH filled by programme graduates.

• Increase the informatics capacity of the public health training system to meet HIV/AIDS information requirements while using an approach that had broad applicability to public health in Zimbabwe.

Interventions to strengthen the MPH programme included a faculty-run curriculum review with technical support from CDC/DGPHCD to ensure that course objectives addressed the needed topics and received adequate focus in the training. Faculty training was provided, both locally and internationally. Modest financial assistance to support retention was provided to departmental faculty working in the MPH programme in return for quality training and mentoring of trainees (difficult to estimate precisely because of currency fluctuations, but representing less than 5% of faculty support). Support was provided both to increase the number of trainees and to improve trainee resources, including computers, textbooks and housing.

A local expatriate technical advisor was hired to assist with teaching and curriculum development and to provide additional technical support for trainees' field projects. An assistant field coordinator, a graduate of the MPH programme, was added to assist in teaching and trainee support and to assist in trainee recruitment. Efforts to increase the number of trainees included providing more resources and increasing recruitment among health professionals by the newly hired staff.

Expansion of HIV/AIDS training in the curriculum was addressed by collaborating with many of the persons and agencies involved in HIV/AIDS in the country to create an HIV interventions course tailored to Zimbabwe's situation. National leaders from major HIV-related programmes lectured trainees. Exercises were conducted to place trainees in the position of designing appropriate HIV interventions based on local data and in the context of Zimbabwe's actual programme constraints and opportunities. As a resource for trainees to use during and after the course, all core national HIV/AIDS policy, strategy and programme documents were collected and put on a CD-ROM for trainees.

The trainees in applied epidemiology spend 14 months in the field applying the public health lessons they have learnt during their classroom instruction. They are required to complete five service learning activities at their field site. The topics addressed by the trainees are chosen based on local priorities. They are expected to apply the knowledge and skills from the HIV course to local issues in the provinces and cities where they conduct their field work. 

The expansion of computer training and capacity in the MPH programme, the university and the MOH was accomplished through a number of activities. Funds were provided to ensure that each MPH trainee had a laptop computer. Computer training was provided to MPH trainees and others by means of a newly created training lab with 36 computer stations. E-mail access was expanded so that all provinces had access to e-mail through a toll-free number without requiring Internet browser access. In addition, the e-mail programme was improved so attachments could be mailed easily, something that had been very difficult in the past.

## Results

CDC committed approximately USD 400 000/year to this programme, or approximately 5% of its then USD 8 million annual budget for Zimbabwe. The level of support remained relatively constant for the years encompassed by this manuscript (2001–2006). In the nearly six years since the programme began, several important changes and indicators support the success of the programme. Table 1 summarizes these changes.

**Table 1:** Goals and achievement of an intervention to strengthen epidemiological and public health leadership training in the Zimbabwe Field Epidemiology Training Programme, 2001–2006

**Goals**	**Status: pre-intervention**	**Status: post-intervention**
Strengthen the public health leadership training programme and increase its output	Curriculum linked to faculty availability 4–8 graduates/year	Revised, standardized curriculum with electronic materials available to faculty and students 10–16 graduates/year (see Table 2)

Increase the focus on training for HIV intervention programmes	No HIV-specific course Few HIV-specific projects	1-week-long course "Responding to HIV" Increased number and proportion of HIV projects (see Table 3)

Increase the number of HIV and related positions in the MOH filled by programme graduates	3 HIV-related positions in MOH filled by graduates 2 HIV-related positions outside MOH held by graduates	7 HIV-related positions in MOH filled by graduates 4 HIV-related positions outside MOH held by graduates (as of 2005)

Increase the informatics capacity of the public health training system	Limited Internet outside the capital city Critique of field assignees work through postal shipment of hardcopy comments	Regular email and Internet at Provincial Medical Directorates Establishment of computer training lab Critique of field assignees work through Track Changes in emailed attachments

### Goal 1. Strengthen the public health leadership training programme and increase its output

The curriculum review led to restructuring of the epidemiology course and clarifying of course objectives. A CD-ROM was created for the course lectures and teaching materials to serve as a resource for trainees and faculty.

Since the strengthening process began, an increased number of trainee projects have been accepted for presentation at international conferences. In the first eight years of the programme, five papers had been accepted for presentation at the CDC Epidemic Intelligence Service Conference. In the past four years, seven papers have been presented. Also, at least eight manuscripts from trainees are being prepared or have been submitted for publication from the last three years, compared with one for the three years preceding this programme. In addition, whereas most trainee work had previously been published only in regional journals, manuscripts are now being submitted to international journals.

Associated with the difficult economic climate in Zimbabwe, overall in the Faculty of Medicine at UZ, as of June 2004, only 133 (43%) of 307 available positions were filled, primarily due to the departure of staff from Zimbabwe. In contrast, the faculty staffing in the Department of Community Medicine had nearly a full complement, with 13 (76%) of 17 posts filled. The number of trainees graduating from the programme has increased since the intervention (Table 2).

**Table 2:** Number of trainees in University of Zimbabwe MPH programme, by year

**Intake years**	**Number of trainees**
1993–1994	8

1995–1996	12

1997–1998	13

1999–2000	13

2001–2002	17

2003–2004	25

2005–2006	23

### Goal 2. Increase the focus on training to produce public health leaders explicitly equipped to design and implement HIV intervention programmes

A one-week HIV/AIDS course (Responding to the Epidemic) was created and was delivered to the MPH trainees beginning in 2002. The course goals were to provide practical knowledge about key evidence-based technical strategies for HIV prevention and care, orientation to key national frameworks for response to HIV/AIDS, an assessment of the HIV/AIDS situation, and skills in monitoring and evaluation of HIV/AIDS interventions. Ultimately, the course aimed to empower trainees to help implement and manage health-sector interventions for HIV/AIDS. The one-week HIV course has now been incorporated as a standard part of the MPH curriculum.

Evaluation after the first year led to greater involvement of the trainees' field supervisors. For the second and subsequent years, the field supervisors were oriented to the training; several supervisors (all Provincial Medical Directors) were asked to participate in the planning and presentation of the HIV course for the trainees.

The resource CD created for the HIV course incorporating a number of review and reference materials was well received and used. In the six-month post-course evaluation, approximately 80% (19 of 23) of the trainees reported using these materials in their field assignments for teaching in provincial and district level courses and in the community, and to provide reference material for provincial and district projects on various HIV interventions.

During the three years since the course began, there has been a substantial increase in the number of HIV-related trainee projects (Table 3). However, the great majority (>80%) of projects still are drawn from the full range of non-HIV/AIDS topics, so that the increased focus on AIDS has not resulted in a restricted scope for the exposure of trainees.

**Table 3:** Number and proportion of trainee HIV-related field projects before and after HIV course intervention

**Intake years**	**Trainees**	**Total field projects**	**HIV-related project (%)**	**Percent of trainees with at least one HIV-related project**
1993–2001	46	241	8 (3.3%)	13%

2002–2004	38	190	26 (13.7%)	58%

During 1993–2000, of 41 MPH dissertation projects completed, three (7%) were HIV-related. From 2001–2004, 13 (34%) of 38 dissertation topics studied HIV-related programmes. Examples of other HIV-related trainee projects and their impact on policy and practice in Zimbabwe include:

• a study of infant feeding among HIV-positive mothers in 2003 that led to increased training in feeding counselling for PMTCT staff;

• an evaluation of a commercial sex worker (CSW) peer education programme in 2003 that led to programme expansion and the development of a new Sexually Transmitted Infection clinic for CSWs in Chinoyi;

• a study on treatment outcomes for patients on antiretroviral therapy (ART) in Bulawayo 2004 that demonstrated favourable outcomes among patients on ART and high adherence levels;

• a study of adverse events and adherence to Highly Active Antiretroviral Therapy (HAART) in Harare that led to clinicians' adopting a modified form of the ACTG grading system on adverse events to guide them in managing adverse events and to switch therapy as appropriate;

• a study on factors associated with non-adherence to HAART in Harare, 2006, that facilitated the opening up of dialogue on coordination of activities by private doctors and the city health doctors concerning ART.

### Goal 3. Increase the number of HIV and related positions in the MOH filled by programme graduates

The expansion of HIV resources, from both the CDC GAP programme and other programmes, public and private, has created a number of positions that require well-trained public health professionals prepared to develop, run and evaluate HIV/AIDS-intervention programmes. Graduates from the MPH programme have been hired for a number of these positions. As of September 2005, HIV/AIDS-related positions in the MOH filled by MPH graduates include Director, National Department of Disease Prevention and Control; Director, PMTCT Programme; Director, ARV Treatment Programme; TB Manager, AIDS and TB Unit; Workplace Officer, AIDS and TB Unit; Training Officer, AIDS and TB Unit; and ANC Surveillance Officer.

MPH graduates in HIV/AIDS positions with other organizations include WHO HIV/AIDS Officer for Zimbabwe; the lead programme officer for HIV Care at United States CDC in Zimbabwe; the Senior Technical Officer for USAID's ARV Treatment Program; and programme officer for HIV projects at UZ-UCSF Research Program. Training in this programme has made the graduates attractive for both MOH positions as well as other public health positions in the country; 30 of 35 recent graduates (2000–2003) are employed in public health positions in Zimbabwe.

### Goal 4. Increase the informatics capacity of the public health training system to meet HIV/AIDS strategic information requirements while using an approach that has broad applicability to public health in Zimbabwe

The computer laboratory has been established and used to teach EpiInfo [24]; the WHO HIV/AIDS Epidemic Projection Package [25]; the International Computer Drivers License [26]; and other software packages to MPH trainees, faculty, Ministry staff and other persons. The expansion of e-mail access to all provinces and the easy use of attachments have greatly facilitated the interaction of trainees in the field with faculty supervisors assisting with their applied learning and research projects. This has allowed rapid feedback to trainees on their proposals, assistance with data analysis through sharing of data files, and assistance with manuscript preparation, predominantly through the Track-Changes features of word-processing software.

In the past, trainees often had to wait for the regular mail system to send and receive hardcopy comments on their fieldwork. In addition, the strengthening of the telecommunications infrastructure (especially more reliable and efficient e-mail) has facilitated the ongoing technical support for the trainees from an expatriate technical advisor who is no longer resident and has substantially improved communication with other technical experts outside Zimbabwe. Moreover, because this strengthened communication capacity now exists at provincial health departments where the trainees' core field training takes place, it has directly facilitated expansion of HIV programmes by supporting distance-based technical support for HIV prevention and treatment programmes and sharing of files and reports for monitoring and evaluation.

## Discussion

This programme demonstrates the ability of the CDC Global AIDS Program in Zimbabwe to invest a relatively small proportion of its HIV programme-specific funds to effectively and substantially help to expand the public health leadership in key positions. Moreover, rather than create a detached HIV training programme, CDC GAP collaborated with multiple partners to strengthen an existing critical component of the public health capacity-building system that in turn directly assists the MOH and CDC to meet HIV-specific programme goals. The key element of this vision was the appreciation that achieving HIV/AIDS programme goals could be realized through catalytic support of general public health leadership capacity development, and did not require narrow HIV-specific training programmes.

We are not able to attribute the rise in HIV-related trainee projects solely to the increased HIV coursework. The number of projects also increased at a time of increased HIV resources and activities in the public health sector. However, this is exactly the type of outcome desired, as the curriculum was designed to reflect the actual health priorities and burden of disease in the country. This programme thus addresses the identified need to develop public health curricula that reflect the emerging needs of the health system where the trainees will work after training [8].

Much of the strength and effectiveness of the programme comes from the fact that others had invested in public health capacity strengthening several years before [10,27]. The existence of the public health training programme and its integration into the public health infrastructure allowed the new resources to strengthen and expand the system for a more rapid and widespread effect. Reinforcing existing and fundamental local institutions, rather than developing a parallel system to train HIV programme leaders, is likewise an important strategy for promoting long-term sustainability.

Many persons who leave developing countries for higher education programmes do not return to their country of origin [28]. In-country, applied training programmes both keep dedicated, trained health workers in the country and allow trainees to contribute immediately and productively to important public health issues while still in training and in formal mentoring [10,19,21,22].

Programme evaluations have found that trainees and graduates of applied epidemiology training programmes form solid networks in the country's health systems, with the majority of them remaining in public health in their home countries [18,20,22,29]. A recent review of the Central American FETP demonstrated the same high level of retention and placement of graduates in public health leadership positions [30].

Given the current economic and political difficulties in Zimbabwe [31-33], retaining well-trained staff is a serious concern. However, this programme has documented its capacity to help train new public health leaders who are already experienced in working on in-country problems and programmes and – judging from the retention rates observed – who appear committed to staying.

The higher retention of faculty for the programme compared with the other departments in the Faculty of Medicine may be partially related to the modest support and training provided, but could also be related to differential external opportunities for the non-clinical faculty in the Department of Community Medicine. These resources are no longer being provided and may not be crucial to programme success.

Evaluation of similar programmes has shown that the managers and decision-makers report numerous examples of how information from trainees and graduates was valuable to them in designing and implementing health programmes, e.g. introducing rubella vaccine and hepatitis B vaccination programmes in Thailand after FETP investigations [29]. The success of the programme in Zimbabwe and similar programmes has led to renewed interest in developing similar programmes across Africa [21]. New field epidemiology training programmes are now being developed in Ethiopia, Nigeria, South Africa, Tanzania and in western Africa. These are being supported in a similar fashion with "vertical" (mostly HIV/AIDS and pandemic influenza) funding. They are using vertical funding sources to produce disease-specific results while also contributing "horizontally" to overall public health system strengthening by building capable public health leaders, adopting the so-called diagonal approach [34].

## Conclusion

This report provides an example of how investment of a modest proportion of new HIV/AIDS resources in targeted public health leadership training programmes can assist in building human capacity to lead and manage national HIV and other public health programmes.

As donors seek to expand programmes to address global disease concerns, including the HIV epidemic, access to well-trained staff and supportive and collaborative ministry officials will be essential. Investment in well-trained staff and emerging programme leaders will be essential to addressing absorption capacity for the medium term while also addressing short-term emergency needs. This model of linking public health leadership capacity building to the HIV/AIDS programme goals provides one example for intervention in this area.

## Competing interests

The authors declare that they have no competing interests.

## Authors' contributions

The paper was conceived by DJ and written with participation from all authors. The original project was conceived by MSL, PN and MT. GW, SH and NS, together with all the other authors, participated in the programme implementation and management. All authors have reviewed and commented on drafts, and have seen and approved the final version.

## Authors' information

DJ is a Medical Epidemiologist, Division of Global Public Health Capacity Development (previously Division of International Health), CDC. MT is Field Coordinator, MPH Programme, Department of Community Medicine, University of Zimbabwe Faculty of Medicine. GW is a Senior Research Epidemiologist, RTI International (formerly Chairman, Department of Community Medicine, University of Zimbabwe Faculty of Medicine). PN is a Medical Epidemiologist, Division of Global Public Health Capacity Development (previously Division of International Health), CDC. NS is a Health Communication Specialist, Global AIDS Program, CDC. SH is Senior Deputy Director, HIV/AIDS Administration (formerly Director, CDC Zimbabwe). MSL is a Senior Science Officer, Coordinating Office for Global Health, CDC (formerly Director, CDC Zimbabwe).
